# Nuclear IDH3A Drives Transcriptional Programs in Melanoma via the YBX1–JUN/FOS Axis

**DOI:** 10.1002/advs.77762

**Published:** 2026-09-11

**Authors:** Juan Ran, Yanchun Fang, Shengming Ruan, Juling Wang, Songbai Liu, Yuan Mao, Dongsheng Hou, Yanping Wang, Zi Ye, Qiong Li, Daoxiang Zhang

**Affiliations:** ^1^ School of Basic Medical Sciences Anhui Medical University Hefei China; ^2^ Department of Pathology Nantong Haimen District People's Hospital Nantong China; ^3^ School of Life Sciences Anhui Medical University Hefei China; ^4^ Chongqing Banan Traditional Chinese Medicine Hospital Chongqing China; ^5^ Jiangsu Province Engineering Research Center of Molecular Target Therapy and Companion Diagnostics in Oncology Suzhou Vocational Health College Suzhou China; ^6^ Hefei Kingmed Medical Laboratory Hefei Anhui China; ^7^ School of Biological Sciences and Medical Engineering Southeast University Jiangsu China; ^8^ Department of Pathology Shandong Provincial Hospital Affiliated to Shandong First Medical University Jinan Shandong China; ^9^ Institute of Hydrobiology Chinese Academy of Sciences Wuhan China; ^10^ Department of Laboratory Medicine Renji Hospital, School of Medicine, Shanghai Jiao Tong University Shanghai China

**Keywords:** IDH3A, melanoma, NONO, YBX1

## Abstract

Isocitrate dehydrogenase 3 alpha (IDH3A) is a key rate‐limiting enzyme in the tricarboxylic acid (TCA) cycle, traditionally associated with cellular energy metabolism. However, its role in cancer remains incompletely understood. Here, we demonstrate that IDH3A is significantly overexpressed in melanoma, primarily due to DNA copy number amplification. Metabolomic analysis revealed that IDH3A overexpression enhanced multiple biosynthetic intermediates, including G6P, F6P, DHAP, and 6‐phosphogluconate, indicating metabolic reprogramming toward anabolic processes without significantly affecting ATP or lactate production. Intriguingly, IDH3A localizes to the nucleus in melanoma cells and promotes tumorigenesis independent of its dehydrogenase activity. Nuclear IDH3A interacts with transcription factor YBX1, enriching at promoter regions of oncogenes such as c‐FOS and c‐JUN, thereby suppressing apoptosis and promoting tumor growth. Furthermore, we identify NONO as a nuclear chaperone that facilitates the nuclear localization of IDH3A through direct interaction, primarily involving NONO amino acids Q166 and S207. Disruption of NONO impairs IDH3A nuclear translocation and mitigates its tumor‐promoting function. Collectively, our findings uncover a noncanonical role of IDH3A as a nuclear regulator of transcription via YBX1, offering novel insight into metabolic enzyme reprogramming in melanoma.

## Graphical abstract

Genomic amplification drives aberrant nuclear localization of IDH3A in melanoma. Independent of its canonical metabolic activity, nuclear IDH3A cooperates with YBX1 to activate the c‐JUN/c‐FOS transcriptional program, while NONO facilitates its nuclear localization. This non‐canonical signaling axis promotes melanoma progression and reveals a previously unrecognized therapeutic vulnerability.

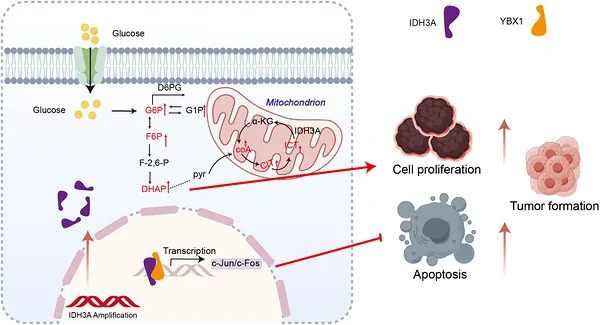

## 1 Introduction

Melanoma is a highly malignant skin cancer with poor prognosis in advanced stages [1]. Despite the success of targeted therapies and immune checkpoint blockade, a substantial proportion of patients develop resistance, highlighting the urgent need to identify novel mechanisms driving melanoma progression [2]. Metabolic reprogramming has emerged as a hallmark of cancer, enabling tumor cells to sustain proliferation and evade cell death [3]. However, the contribution of specific metabolic enzymes beyond their canonical roles remains incompletely understood.

One emerging hallmark of cancer is metabolic reprogramming, wherein tumor cells rewire central metabolic pathways to support anabolic growth and adapt to environmental stress [4]. While much attention has been given to well‐established metabolic enzymes such as IDH1 and IDH2—particularly in glioma and leukemia where their oncogenic mutations lead to production of the oncometabolite 2‐hydroxyglutarate [5, 6, 7, 8], the role of IDH3A, the catalytic subunit of the mitochondrial NAD^+^‐dependent IDH3 complex [9], has remained largely unexplored in solid tumors. Unlike IDH1/2, IDH3A is rarely mutated; however, emerging studies suggest it may be overexpressed in cancers such as glioblastoma and hepatocellular carcinoma, and potentially implicated in metabolic adaptation [10, 11]. Whether IDH3A acts as a driver of melanoma progression, and if so, through what mechanisms, remains unknown. Interestingly, recent studies suggest that mitochondrial enzymes can translocate to the nucleus and exert noncanonical regulatory functions, particularly in development and cancer. Whether IDH3A possesses such “moonlighting” activity in melanoma is currently unknown.

Here, we identify IDH3A as a copy number–driven oncogene frequently overexpressed in melanoma. Unexpectedly, we discover that IDH3A translocates to the nucleus, where it promotes tumor growth independently of its classical enzymatic function. Mechanistically, nuclear IDH3A interacts with the transcription factor YBX1 and is recruited to the c‐JUN and c‐FOS promoters in a YBX1‐dependent manner, enhancing their expression and suppressing apoptosis. Disruption of YBX1 abrogates the transcriptional and oncogenic effects of IDH3A, establishing a critical IDH3A–YBX1–AP1 axis. Furthermore, we uncover the RNA‐binding protein NONO as a key mediator of IDH3A nuclear localization via direct interaction, and show that NONO loss impairs the nuclear function and tumorigenic potential of IDH3A.

Collectively, our study reveals a previously unrecognized nonmetabolic role of IDH3A in melanoma, functioning as a nuclear transcriptional co‐regulator that drives tumor progression through the YBX1–AP‐1 pathway. These findings expand the paradigm of metabolic enzyme reprogramming and uncover potential vulnerabilities in melanoma amenable to therapeutic intervention.

## 2 Materials and Methods

### 2.1 Cell Culture

Human melanocytes (HEM) were obtained from iCell Bioscience (Shanghai, China), and human melanoma cell lines A375(RRID:CVCL_6233), A875(RRID:CVCL_4733), A2058(RRID:CVCL_1059), MV3(RRID:CVCL_W280), as well as HEK293T(RRID:CVCL_0063)cells, were kindly provided by Dr. Xiaoying Liu (Anhui Medical University). Cell lines were authenticated by the provider using STR profiling. All cell lines were routinely tested and confirmed to be free of mycoplasma contamination before use. All cell lines were maintained in high‐glucose DMEM (Gibco or Lanjieke Biosciences) supplemented with 10% fetal bovine serum (FBS) and 1 mM sodium pyruvate at 37°C in a humidified incubator with 5% CO_2_. Detailed information on antibodies and key reagents is provided in Table S2.

### 2.2 Plasmid Construction

To generate Flag‐tagged IDH3A and YBX1 expression constructs, cDNAs encoding full‐length human IDH3A and YBX1 were PCR‐amplified from MV3 cells and cloned into the BsiWI and MluI sites of the pHR‐sin‐GFP‐Puro vector. The IDH3A catalytically impaired IDH3A‐Y153F mutant (hereafter referred to as IDH3A‐mut) and the mitochondria localization sequence‐deleted form (IDH3A‐dMTS) were constructed via two‐step PCR‐based mutagenesis, and DpnI digestion was used to eliminate template DNA. For shRNA constructs, oligonucleotide pairs targeting IDH3A or YBX1 were annealed and cloned into the EcoRI and XhoI sites of the PGIPZ vector.

### 2.3 Generation of Stable Cell Lines

Lentiviral particles were produced in HEK293T cells using a three‐plasmid packaging system and PEI‐mediated transfection. MV3 and A2058 cells were infected with lentiviruses encoding IDH3A‐WT, IDH3A‐mut, or IDH3A‐dMTS, while A2058 and A375 cells were infected with lentiviruses expressing shIDH3A. Stable cell lines were selected using puromycin (2 µg/mL), and successful expression or knockdown was verified by immunoblotting.

### 2.4 Transient Transfection

Transient transfection was performed using polyethyleneimine (PEI) in 6‐well plates. A2058 and A375 cells were transfected with siRNAs targeting IDH3A (50 nM final concentration), and MV3 cells stably expressing vector control, IDH3A‐WT, IDH3A‐mut, or IDH3A‐dMTS were transfected with siYBX1 (50 nM). Cells were harvested 48–72 h post‐transfection for subsequent analysis.

### 2.5 Colony Formation Assay

To assess clonogenic capacity, 500 cells per well were seeded in 6‐well plates. MV3 and A2058 cells stably expressing vector control, IDH3A‐WT, IDH3A‐mut, or IDH3A‐dMTS were cultured for 12 days. A2058 and A375 cells transfected with shIDH3A or siIDH3A were treated similarly. Colonies were fixed and stained with crystal violet (0.5% in methanol) for 20 min, rinsed with distilled water, and counted under a stereomicroscope.

### 2.6 Soft Agar Colony Formation Assay

Anchorage‐independent growth was assessed using a two‐layer soft agar assay. A bottom layer of 0.6% agar (prepared by mixing 2.4% agar stock with complete medium) was poured into 24‐well plates (500 µL/well). Approximately 500 cells were suspended in 0.3% agar and seeded onto the bottom agar layer. Colonies were allowed to grow for 12 days and were manually counted under a light microscope. Three independent wells were analyzed for each group.

### 2.7 RT‐qPCR

Total RNA was isolated using RNA Extraction Reagent (AN51L758, Shanghai Leji Biotech) according to the manufacturer's protocol. Reverse transcription was performed using a commercial RT kit (11142ES60, Yeasen Biotech). Quantitative PCR was carried out using SYBR Green Master Mix (Applied Biosystems) on the StepOnePlus Real‐Time PCR System. Relative gene expression was calculated using the 2^–ΔΔCt method. Sequences are listed in Table S1.

### 2.8 Immunoblotting

Total protein lysates were prepared by lysing cells in RIPA buffer (Lanjieke, Beijing) supplemented with protease inhibitors (Yeasen Biotech) on ice for 10 min, followed by centrifugation at 12 000 rpm for 15 min at 4°C to remove insoluble debris. Supernatants were collected and subjected to SDS‐PAGE, and proteins were transferred onto nitrocellulose membranes (Junyi). Membranes were incubated with primary antibodies diluted according to manufacturers’ instructions, followed by incubation with HRP‐conjugated secondary antibodies. Immunoreactive bands were visualized using an enhanced chemiluminescence (ECL) detection system (Tanon, Shanghai) and quantified with ImageJ software.

Primary antibodies used included: IDH3A (Rabbit, 15909‐1‐AP), Tubulin (66031‐1), PARP1 (13371) from Proteintech; GAPDH (sc‐47724), Ku‐86 (sc‐5280), c‐Jun (sc‐74543), c‐Fos (sc‐166940) from Santa Cruz Biotechnology; Melan‐A (AB0152701) from DUONENG‐BIO; Caspase‐3 (14220), Cleaved Caspase‐3 (9664) from Cell Signaling Technology; YBX1 (EP2708Y) from Abcam; IDH3A (Mouse, TA500739) from Origene. HRP‐conjugated secondary antibodies included anti‐rabbit (PR30011) and anti‐mouse (PR30009).

### 2.9 Immunohistochemistry (IHC)

Human melanoma specimens and matched non‐tumor skin tissues were obtained from Qilu Hospital of Shandong University between 2021 and 2022. A total of eight melanoma specimens and eight matched non‐tumor skin specimens were included in this study. Paraffin‐embedded sections of human melanoma tissues were deparaffinized, rehydrated, and subjected to antigen retrieval. After blocking with goat serum, sections were incubated with antibodies against IDH3A, c‐FOS, or c‐JUN, followed by HRP‐conjugated secondary antibody incubation and detection with 3,3'‐diaminobenzidine (DAB). Sections were counterstained with hematoxylin. All reagents were obtained from Beyotime Biotechnology (Shanghai, China). Immunohistochemical staining was independently evaluated by two experienced pathologists who were blinded to the clinicopathological information and experimental grouping. Nuclear IDH3A staining was quantified using the H‐score method, calculated by multiplying the percentage of positively stained cells by the corresponding staining intensity (0 = negative, 1 = weak, 2 = moderate, and 3 = strong), yielding a total score ranging from 0 to 300.

### 2.10 Fluorescence In Situ Hybridization (FISH)

To detect IDH3A DNA copy number in melanocytes and melanoma cells, HEM, MV3, and A2058 cells were seeded onto sterile coverslips. Cells were fixed with methanol and permeabilized, followed by co‐denaturation of probe and genomic DNA. Slides were subjected to ethanol dehydration gradient, hybridized with a FAM‐labeled IDH3A probe overnight at 37°C, and subsequently washed and counterstained with DAPI. Antifade mounting medium was added, and slides were imaged using a laser confocal microscope. At least 50 cells per group were analyzed, and quantification was performed using GraphPad Prism software.

The Sequence of the IDH3A DNA Probe Was:5’‐FAM‐tgacttgtgtgcaggattgatcggaggtctcggtgtgacaccaagtggcaacattggagccaatggggttgcaatttttg‐3’.

### 2.11 Digital Droplet PCR (ddPCR)

To quantify the genomic abundance of IDH3A in melanoma cell lines and clinical melanoma tissue specimens, genomic DNA was extracted using the TIANamp Genomic DNA Kit (Tiangen Biotech) according to the manufacturer's instructions. DNA concentration was measured using a NanoDrop spectrophotometer and diluted to a uniform working concentration with nuclease‐free water. Droplet digital PCR (ddPCR) was performed using the Bio‐Rad QX200 Droplet Digital PCR system following the manufacturer's protocol. Reactions were prepared with ddPCR Supermix for Probes (No dUTP, Bio‐Rad), specific primers and probes targeting IDH3A, and 20 ng of genomic DNA per reaction. Droplets were generated using the QX200 Droplet Generator and subjected to PCR amplification under optimized cycling conditions. Droplet fluorescence was read using the QX200 Droplet Reader, and data were analyzed with QuantaSoft software (Bio‐Rad). QuantaSoft software calculated the absolute copy number (copies/µL) for each sample. The resulting absolute copy numbers were subsequently normalized to the HEM group and are presented as relative IDH3A genomic copy number. Only reactions with >12,000 accepted droplets were included for analysis. Three independent biological replicates were analyzed, and the results were presented as the relative genomic abundance of IDH3A normalized to the HEM group. The sequences of the IDH3A‐specific primers and TaqMan probe used for ddPCR were as follows: IDH3A‐forward primer: 5′‐ACATTCTTTGAAATGCTTTCT‐3′; IDH3A‐reverse primer: 5′‐ACACGCTGTTCTGACCTGAGTT‐3′; IDH3A probe: 5′‐FAM‐CCAAGTGGCAACATTGGAGC‐BHQ1‐3′. The expected amplicon size was 162 bp.

### 2.12 Genomic Quantitative PCR

Genomic DNA was extracted using the TIANamp Genomic DNA Kit. IDH3A genomic abundance was independently assessed by quantitative PCR using a primer set distinct from that used for ddPCR. ACTB was used as the reference genomic locus. Relative IDH3A genomic abundance was calculated using the 2^−ΔΔCt method. Primer sequences are provided in Table S1.

### 2.13 Chromatin Immunoprecipitation–Quantitative PCR (ChIP–qPCR)

ChIP–qPCR was performed using a standard protocol. Briefly, cells were crosslinked with 1% formaldehyde, and chromatin was sonicated to generate DNA fragments of approximately 200–500 bp. Chromatin was immunoprecipitated overnight at 4°C with an anti‐IDH3A antibody or normal rabbit IgG as a negative control. After reversal of crosslinking and DNA purification, enriched DNA was analyzed by quantitative PCR using primers targeting the c‐JUN and c‐FOS promoter regions. ChIP signals were normalized to the input DNA, and all experiments were performed with at least three independent biological replicates.

### 2.14 Molecular Docking and Molecular Dynamics Simulation

The docked complex was subjected to molecular dynamics simulations using GROMACS 2022.3 with the Amber99SB‐ILDN force field. The system was solvated in a TIP3P water box and neutralized by adding appropriate counterions. After energy minimization and equilibration under NVT and NPT ensembles, a 100‐ns production simulation was performed. Structural stability was evaluated by RMSD, RMSF, radius of gyration (Rg), solvent‐accessible surface area (SASA), hydrogen bond analysis, and MM/PBSA binding free‐energy calculations.

### 2.15 Targeted Metabolomics Analysis

Cell samples were prepared according to the sample submission requirements of Shanghai Bioprofile Biotechnology Co., Ltd. Cells were collected in triplicate per group, pelleted, snap‐frozen in liquid nitrogen, and stored on dry ice prior to shipment for targeted metabolite profiling. Energy metabolism metabolites in MV3‐Control and MV3‐IDH3A overexpression (OE) cells were quantitatively analyzed.

### 2.16 ATP Measurement

Melanoma cell lines MV3 and A2058 (Control, IDH3A‐WT, IDH3A‐mut, and IDH3A‐dMTS) were seeded in 6‐well plates. When cells reached ∼80% confluence, ATP levels were measured using the ATP Assay Kit (Shanghai BioCloud, Cat No. S0026) following the manufacturer's instructions. Luminescence was recorded on a GloMax Navigator microplate reader (Promega). Each condition was tested in triplicate, and data were expressed as the mean ± SD.

### 2.17 Glucose Quantification

Cells were seeded as described above. At ∼80% confluence, glucose content was assayed using a glucose detection kit based on the O‐toluidine method (Shanghai BioCloud, Cat No. S0201S). Absorbance was measured on a multifunctional microplate reader. Experiments were performed in triplicate, and results were averaged.

### 2.18 Lactate Quantification

Lactate levels were measured in the same cell groups using a lactate assay kit (Sangon Biotech, Cat No. D799099) according to the manufacturer's protocol. Measurements were conducted on a multifunctional microplate reader, with triplicate samples analyzed and averaged.

### 2.19 Seahorse XF Metabolic Flux Analysis

MV3‐Control, MV3‐IDH3A‐WT and A375‐ShIDH3A (sh1 and sh3) cells were trypsinized, counted, and seeded at 80,000 cells per well in Seahorse XF24 plates. Cells were allowed to settle for 1 h at room temperature and then incubated overnight at 37°C. Mitochondrial respiration assays were performed by the Seahorse instrument facility at Anhui Medical University according to standard protocols. Data were analyzed using GraphPad Prism.

### 2.20 Oil Red O Staining

Frozen melanoma tissue sections (15 µm thickness) were stained with Oil Red O (Shanghai BioCloud, Cat No. C0157S) per the manufacturer's instructions to assess lipid accumulation associated with IDH3A expression. Images were captured under an inverted phase‐contrast microscope. Lipid‐positive areas were quantified in three randomly selected high‐power fields per section, and results were averaged.

### 2.21 Nuclear and Cytoplasmic Protein Extraction

Cell pellets from MV3 and A2058 cells were lysed in ice‐cold hypotonic buffer (20 mM Tris‐HCl, pH 7.4, 10 mM KCl, 2 mM MgCl_2_, 0.5 mM DTT, 0.5 mM PMSF), centrifuged, and supernatants collected as cytoplasmic fractions. Nuclear pellets were washed and lysed in isotonic buffer (20 mM Tris‐HCl, pH 7.4, 150 mM KCl, 2 mM MgCl_2_, 0.5 mM DTT, 0.5 mM PMSF) with intermittent vortexing and centrifugation to isolate nuclear proteins. Protein samples were prepared in 6× SDS loading buffer, boiled, and used for immunoblotting.

### 2.22 Transcriptome Sequencing

Total RNA was extracted using TRIzol reagent according to the manufacturer's instructions. The RNA concentration was measured with a NanoDrop 2000 spectrophotometer (Thermo Scientific, USA). RNA sequencing libraries were prepared following the recommended protocol at Nanjing GENEWIZ Biotech Co., Ltd. using the BGI T7 sequencing platform. Transcriptome sequencing and subsequent data analysis were performed through the cloud platform provided by Nanjing GENEWIZ Biotech Co., Ltd. Briefly, raw sequencing reads were subjected to quality control and filtering to remove low‐quality reads and adaptor sequences. Clean reads were aligned to the human reference genome, and gene expression levels were quantified. Differentially expressed genes were identified using DESeq2 with adjusted *p* values calculated by the Benjamini–Hochberg false discovery rate (FDR) correction. The RNA‐seq data generated in this study have been deposited in the Genome Sequence Archive (GSA) under accession number HRA019581.

### 2.23 Immunofluorescence Staining

Cells or frozen tissue sections were fixed, permeabilized, and incubated overnight at 4°C with primary antibodies against IDH3A (Proteintech, Cat No. 15909‐1‐AP), Ki67 (HUABIO, Cat No. HA721115), or Melan‐A (DUONENG‐BIO, Cat No. AB0152701). Appropriate CoraLite‐conjugated secondary antibodies were applied, and nuclei counterstained with DAPI. Fluorescence images were acquired using a Zeiss Axio Observer 3 microscope. Quantification was performed on three independent fields per sample.

### 2.24 Mitochondrial Staining

MV3‐IDH3A‐dMTS and A2058‐IDH3A‐dMTS cells were cultured on glass‐bottom dishes and stained with MitoTracker Red CMXRos (Beyotime, Cat No. C1035) according to the manufacturer's protocol. After DAPI staining, images were acquired by confocal microscopy (Zeiss LSM800 + Airyscan).

### 2.25 Immunoprecipitation‐Mass Spectrometry (IP‐MS)

293T cells were transiently transfected with PHR‐sin‐IDH3A or control vector. Cell lysates were prepared with IP lysis buffer (Beyotime, Cat No. P0013) and incubated with magnetic beads (Beyotime, Cat No. P2115) overnight at 4°C. After washing with 1× TBS, bound proteins were eluted with 3× Flag peptide (Beyotime, Cat No. P9801), snap‐frozen, and sent on dry ice for mass spectrometry analysis (Shanghai Bioprofile Biotechnology Co., Ltd.).

### 2.26 Immunoprecipitation (IP) and Western Blot

293T cells transiently transfected with PHR‐sin, PHR‐sin‐IDH3A, or PHR‐sin‐YBX1, and MV3 variants were lysed and incubated with magnetic beads as described above. Eluted proteins were separated by 10% SDS‐PAGE and transferred onto PVDF membranes. Blots were probed with antibodies against IDH3A (Proteintech), Tubulin (Proteintech), GAPDH (Santa Cruz Biotechnology), and YBX1 (Abcam).

### 2.27 CUT&Tag Assay and Bioinformatic Analysis

CUT&Tag libraries were prepared using the YEASEN CUT&Tag Kit (Cat. No. 12598ES04) according to the manufacturer's instructions. CUT&Tag assays were performed using MV3‐Control and MV3 cells overexpressing FLAG‐tagged IDH3A (MV3‐IDH3A‐WT). Briefly, 1 × 10^5 viable cells from each group were immobilized on Concanavalin A‐coated magnetic beads and sequentially incubated with an anti‐FLAG antibody, secondary antibody, and pA‐Tn5 transposase. Following Mg^2^^+^‐mediated tagmentation, DNA fragments were purified, PCR‐amplified, and subjected to paired‐end sequencing on an Illumina NovaSeq 6000 platform (Nanjing Jisihuiyuan Biotechnology Co., Ltd.).

Raw sequencing reads were quality‐filtered prior to downstream analysis. Adapter and primer sequences were removed, reads containing more than 10% undetermined bases (N) were excluded, and low‐quality reads in which ≥50% of bases had quality scores ≤5 were discarded. After quality filtering, 16 209 154 and 8 722 298 clean reads were obtained for the MV3‐IDH3A‐WT and MV3‐Control samples, respectively, with Q30 values of 86.89% and 84.56%. Clean reads were aligned to the human reference genome (hg38, Ensembl release 104) using Bowtie2 (v2.3.5.1). Only uniquely mapped reads with mapping quality scores greater than 30 were retained for downstream analyses. Peaks were identified using MACS2 (v2.7.16) and annotated with ChIPseeker (v1.26.2). Motif enrichment and transcription factor binding analyses were performed using HOMER (v4.10.4), FIMO (MEME Suite v5.3.0), and the JASPAR 2022 database. Sequencing quality and alignment statistics were evaluated to ensure data quality prior to downstream analyses.

### 2.28 Flow Cytometry Analysis

Apoptosis was assessed in wild‐type MV3 cells and MV3 cells expressing IDH3A‐dMTS. Cells were treated with the AP‐1(c‐JUN/c‐FOS)inhibitor T‐5224 (TopScience, Cat. No. T5416) as indicated, collected, and prepared as single‐cell suspensions. Apoptotic cells were labeled using the Annexin V Apoptosis Detection Kit I (APE BIO, Cat. No. K1133) according to the manufacturer's instructions. Briefly, cells were incubated with Annexin V at room temperature for 30 min in the dark. Labeled cells were analyzed on a CytoFLEX LX flow cytometer (Beckman Coulter), and Data were analyzed using FlowJo software, and the percentages of early and late apoptotic cells were quantified.

### 2.29 GST Pulldown Assay

The gene of interest was cloned into the pGEX vector and transformed into *E. coli* BL21 cells. Expression of GST‐fusion proteins was induced with IPTG for 4–6 h. The fusion proteins were purified using glutathione agarose beads and eluted with elution buffer. Melanoma cells were lysed in lysis buffer supplemented with protease inhibitors to obtain cell lysates. The purified GST‐fusion proteins were incubated with the melanoma cell lysates at 4°C with rotation for 24 h. After incubation, the beads were washed with buffer containing 0.1% Triton X‐100 to remove nonspecific interactions. Finally, bound proteins were eluted by adding sample loading buffer and analyzed by Western blot.

### 2.30 Dual‐Luciferase Reporter Assay

The 3‐kb promoter regions of c‐JUN and c‐FOS were cloned into the PGL3 vector. 293T cells were co‐transfected with 500 ng of PGL3 constructs and IDH3A overexpression plasmid or empty vector. Firefly and Renilla luciferase activities were measured using the Dual‐Luciferase Reporter Assay Kit in triplicate. Mutant constructs targeting YBX1 binding sites in c‐JUN and c‐FOS promoters were generated via site‐directed mutagenesis using specified primers and similarly tested.

### 2.31 TUNEL Assay

Frozen tissue sections were fixed in 4% paraformaldehyde, permeabilized with 0.2% Triton X‐100, and stained with TUNEL reaction mixture at 37°C for 60 min. Sections were counterstained with PI and imaged under a fluorescence microscope. Apoptotic nuclei were quantified in three fields per section.

### 2.32 Melanoma Xenograft Model

All mice were maintained under specific pathogen‐free (SPF) conditions at Anhui Medical University. All animal experimental procedures were approved by the Institutional Animal Care and Use Committee of Anhui Medical University (Approval No. 20231015) and were performed in accordance with relevant institutional guidelines and ethical regulations for animal use. A total of 1 × 10^6^ MV3 melanoma cells with the indicated genetic modifications were subcutaneously injected into the flanks of mice. Mice were randomly assigned to the indicated experimental groups. For pharmacological treatment, T‐5224 was administered by intraperitoneal injection at a dose of 200 mg/kg three times per week. Tumor size was measured every 2–3 days using calipers, and tumor volume was calculated as V = (W^2^ × L)/2, where W represents tumor width and L represents tumor length. At the experimental endpoint, mice were euthanized and tumors were collected for subsequent analyses.

### 2.33 Murine Cutaneous Tumors Model

Female C57BL/6 mice (4–6 weeks old) had dorsal fur shaved prior to chemical induction. DMBA stock solution (25 mg/mL in DMSO) was diluted to 1.5% in anhydrous ethanol and applied topically to the dorsal skin daily for 7 days. Control mice received DMSO/ethanol vehicle. After a 1‐day interval, TPA (200 nmol in ethanol) was applied daily for 4 weeks. Skin lesions were monitored throughout the experiment, and TPA application was continued until cutaneous tumors developed.

### 2.34 Statistical Analysis

Data are presented as mean ± SD unless otherwise indicated. Comparisons between two groups were performed using two‐tailed Student's *t*‐tests, and multiple‐group comparisons were performed using two‐way ANOVA where appropriate. Survival analysis was conducted using the Kaplan–Meier method. Unless otherwise indicated, experiments were performed with at least three independent biological replicates, and technical replicates were included where applicable. For metabolomic analyses, statistical significance was determined using Student's *t*‐test, with multiple‐comparison correction applied where appropriate. All statistical analyses were performed using GraphPad Prism software (version 8.0.2). A *p* value < 0.05 was considered statistically significant.

## 3 Results

### 3.1 IDH3A Promotes Melanoma Cell Proliferation

**FIGURE 1:**
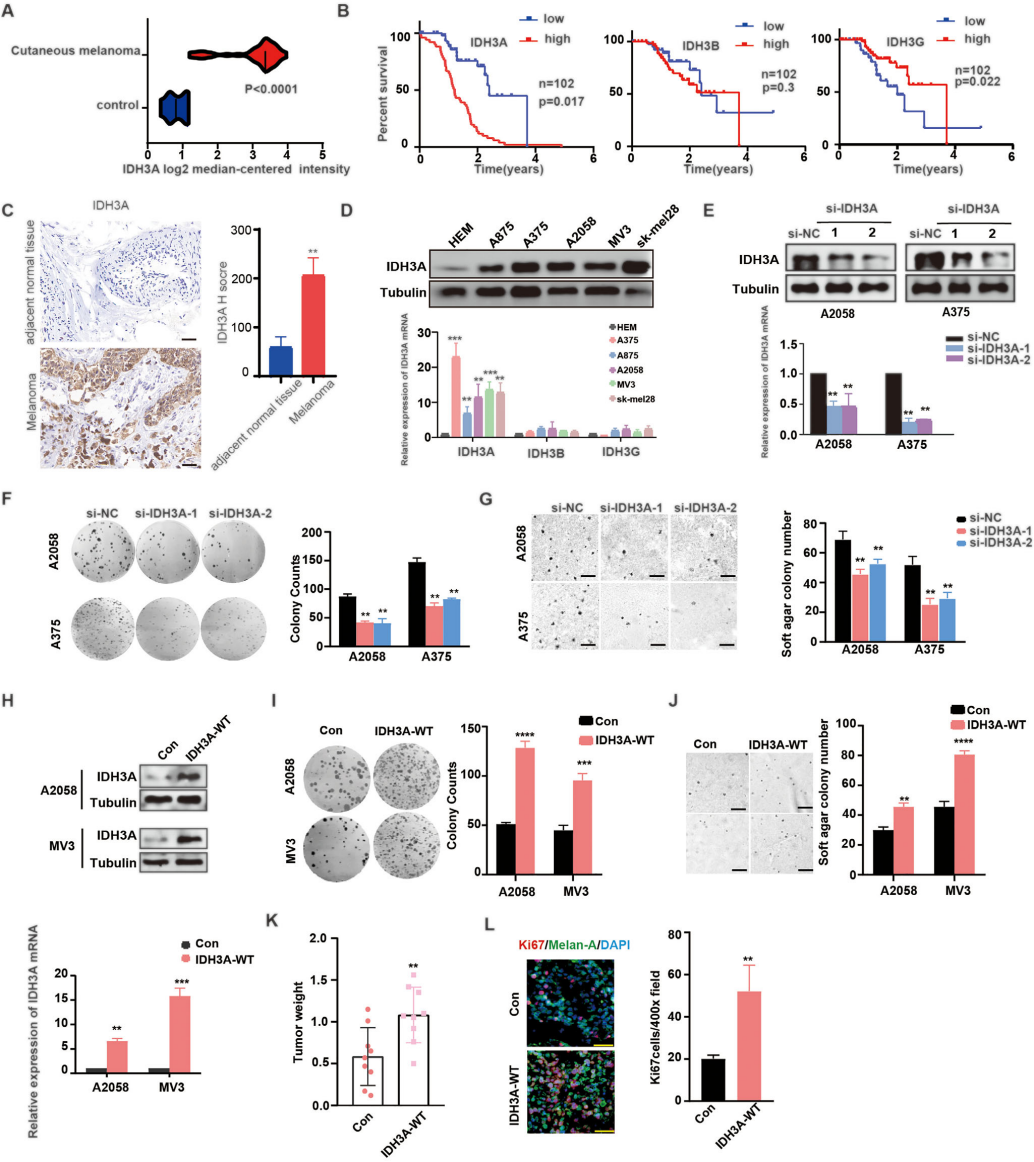
IDH3A promotes melanoma cell proliferation. (A) IDH3A mRNA expression in normal skin (n = 7) and melanoma (n = 45) samples from the publicly available GEO dataset GSE3189. (B) Kaplan–Meier survival analysis based on IDH3A, IDH3B, and IDH3G expression in TCGA melanoma cohorts. (C) Immunohistochemistry (IHC) showing IDH3α expression in melanoma tissues. Scale bars: 10 µm. (D) IDH3A protein and mRNA levels in melanoma cell lines assessed by immunoblotting and qPCR. (E) Efficiency of IDH3A knockdown by siRNAs. (F,G) Colony formation (F) and soft agar (G) assays following IDH3A knockdown. (H) Efficiency of IDH3A overexpression. Scale bars: 100 µm. (I,J) Colony formation (I) and soft agar (J) assays following IDH3A overexpression. (K) Tumor growth in nude mice upon IDH3A overexpression. Scale bars: 100 µm. (L) Immunofluorescence analysis of Ki67 expression in IDH3A‐overexpressing tumor xenografts. Scale bars: 10 µm.

Our previous work has demonstrated that IDH3A is downregulated in cancer‐associated fibroblasts (CAFs) within the melanoma microenvironment, where it contributes to metabolic reprogramming [12]. Interestingly, contrary to its expression pattern in CAFs, analysis of the publicly available GEO dataset GSE3189, together with immunohistochemical analysis of clinical specimens revealed that IDH3A is significantly upregulated in melanoma tissues (Figure 1A,C). Further survival analysis, based on data retrieved from The Human Protein Atlas, indicated that high IDH3A expression is associated with poor prognosis in melanoma patients (Figure 1B). In contrast, expression levels of other subunits of the isocitrate dehydrogenase 3 complex—IDH3β showed no prognostic significance, and high IDH3γ expression correlated with better survival outcomes (Figure 1B). These findings suggest that IDH3A may exert functions independent of the canonical IDH3 enzymatic complex.

To further verify its expression pattern, we performed Western blot analysis on five melanoma cell lines and found that IDH3A protein levels were markedly elevated compared to normal human epidermal melanocytes (HEMs) (Figure 1D). Consistent results were obtained at the mRNA level via qRT‐PCR, while IDH3β and IDH3γ mRNA levels showed no significant changes (Figure 1D). To investigate the functional role of IDH3A in melanoma, we used siRNA‐mediated knockdown in A375 and A2058 cells. Both protein and mRNA levels confirmed efficient silencing by two different siRNAs (siIDH3A‐1 and siIDH3A‐2) (Figure 1E). Functional assays, including colony formation and soft agar assays, demonstrated that IDH3A knockdown significantly suppressed the proliferation capacity of melanoma cells in vitro (Figure 1F,G).

Conversely, stable overexpression of IDH3A via lentiviral transduction in A2058 and MV3 cells resulted in enhanced colony formation and anchorage‐independent growth (Figure 1H–J). To validate these findings in vivo, we established a subcutaneous xenograft model using the MV3 melanoma cell line and found that IDH3A overexpression significantly promoted tumor growth (Figure 1K). IF analysis further confirmed increased Ki67‐positive cells in IDH3A‐overexpressing tumors, supporting its role in cell proliferation (Figure 1L).

Collectively, these data demonstrate that IDH3A is significantly upregulated in melanoma and contributes to tumor proliferation both in vitro and in vivo.

### 3.2 Genomic Amplification Contributes to Elevated IDH3A Expression in Melanoma

Ultraviolet (UV) radiation is a major etiological factor in melanoma, known to cause widespread genomic instability and structural alterations [13]. To investigate the potential genomic basis underlying the elevated expression of IDH3A in melanoma, we analyzed DNA copy number variations using data from The Cancer Genome Atlas (TCGA).

**FIGURE 2:**
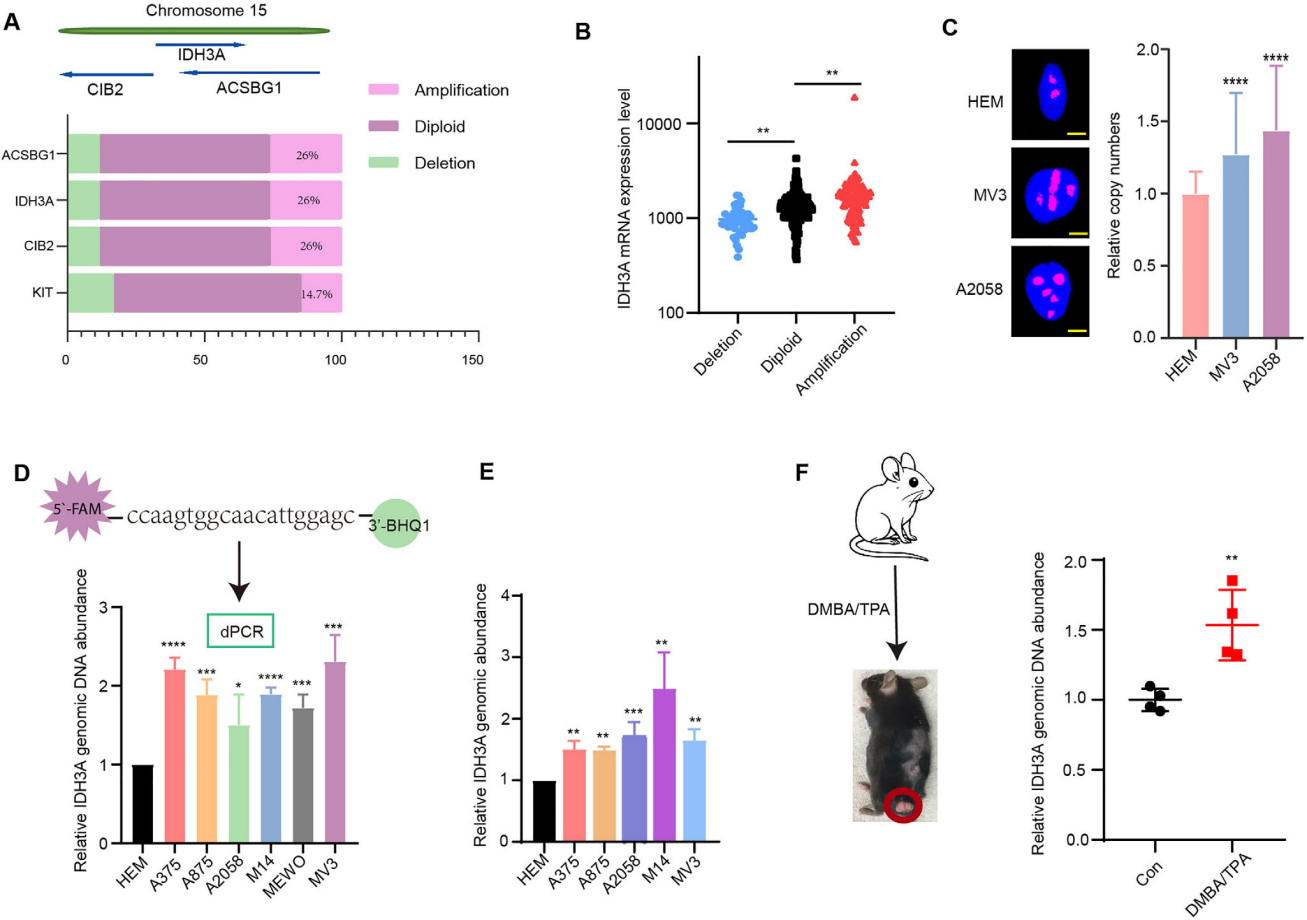
Genomic copy number gain contributes to elevated IDH3A expression in melanoma. (A) Copy number variations of IDH3A and neighboring genes on chromosome 15 in TCGA melanoma samples. (B) Correlation between IDH3A DNA copy number and mRNA expression. (C) FISH analysis of IDH3A copy number in melanoma cells. Scale bars: 5 µm. (D) Absolute quantification of IDH3A DNA copy number by digital PCR in melanoma cell lines. (E) Relative genomic abundance of IDH3A determined by genomic DNA qPCR. (F) Absolute quantification of IDH3A DNA copy number by digital PCR in the DMBA/TPA‐induced mouse model.

Our analysis revealed that IDH3A exhibits copy number increase in approximately 26% of melanoma samples (Figure 2A). Notably, neighboring genes such as CIB2 and ACSBG1 within the same chromosomal locus also showed similar increase frequencies (Figure 2A), suggesting that this genomic region is a hotspot for structural alterations in melanoma. Consistent with previous reports, the KIT oncogene—known for its involvement in melanomagenesis [14] —also showed copy number gains in 14.7% of cases, supporting the prevalence of such genomic events in this cancer type.

Importantly, IDH3A copy number gains were significantly correlated with increased mRNA expression levels, suggesting that increased genomic copy number may contribute to IDH3A overexpression (Figure 2B). To experimentally validate these observations, we performed fluorescence in situ hybridization (FISH) to examine IDH3A genomic copy number in normal human epidermal melanocytes (HEM) and melanoma cell lines (MV3 and A2058). FISH results confirmed that melanoma cells harbor significantly more IDH3A gene copies than normal HEM cells (Figure 2C).

To further quantify the relative IDH3A genomic copy number, we designed a 5’‐FAM‐labeled probe and employed droplet digital PCR (ddPCR) to measure relative IDH3a genomic copy number in six melanoma cell lines. The results consistently showed that melanoma cells showed an increased relative IDH3A genomic copy number compared to HEM controls (Figure 2D). To validate this finding using an independent approach, genomic DNA was extracted from the melanoma cell lines and subjected to quantitative PCR (qPCR). Consistent with the dPCR results, melanoma cells exhibited significantly increased relative genomic copy number of IDH3A compared with HEM cells (Figure 2E).

Moreover, to determine whether the increase in IDH3A copy number observed in human melanoma is also reflected during cutaneous tumorigenesis in vivo, we employed a DMBA/TPA‐induced murine skin tumor model in C57‐background mice. Consistent with the human data, skin tumors generated in this model also exhibited elevated IDH3a genomic copy number (Figure 2F).

Collectively, these data support increased genomic copy number of the IDH3A locus as a potential mechanism contributing to its elevated expression in melanoma.

### 3.3 IDH3A Promotes Melanoma Progression Through Non‐Canonical, Non‐Dehydrogenase Functions

**FIGURE 3:**
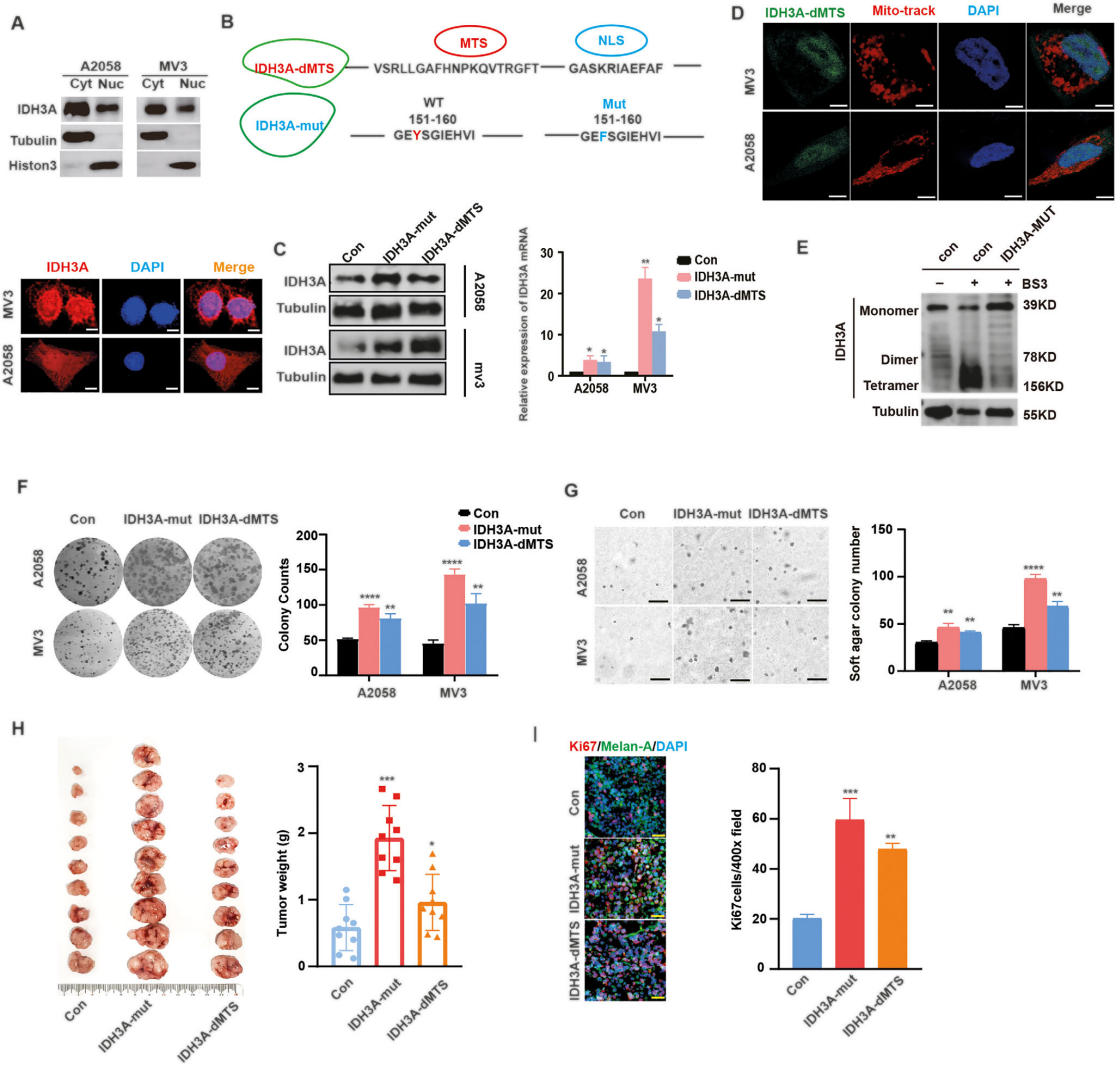
IDH3A promotes melanoma progression via non‐canonical, non‐dehydrogenase functions. (A) Immunoblotting and immunofluorescence showing nuclear localization of IDH3A. Scale bars: 10 µm. (B) Schematic of IDH3A‐dMTS and IDH3A‐mut construct design. (C) Overexpression efficiency of IDH3A‐mut and IDH3A‐dMTS. (D) Subcellular localization of IDH3A‐dMTS. Scale bars: 10 µm. (E) Native PAGE analysis of IDH3A wild‐type and mutant IDH3A. (F and G) Colony formation (F) and soft agar (G) assays with IDH3A‐dMTS and IDH3A‐mut. Scale bars: 100 µm. (H) Tumor formation in nude mice with IDH3A‐dMTS and IDH3A‐mut overexpression. (I) Immunofluorescence staining of Ki67 in xenografts from (H). Scale bars: 10 µm.

Emerging evidence suggests that several metabolic enzymes traditionally confined to the tricarboxylic acid (TCA) cycle can localize to the nucleus during embryonic development, where they perform non‐canonical functions. Given the known parallels between embryonic and tumor cells in terms of metabolic plasticity and proliferation [15, 16], we explored whether IDH3A could also exert nuclear roles in melanoma. Using nuclear–cytoplasmic fractionation assays, we detected the presence of IDH3A in the nuclear compartment of melanoma cells (Figure 3A). Confocal immunofluorescence microscopy further confirmed nuclear localization of IDH3A in both MV3 and A2058 cell lines (Figure 3A).

To investigate the mechanism underlying nuclear localization, we analyzed the IDH3A amino acid sequence and identified a classical nuclear localization signal (NLS), GASKRIAEFAF, which is highly conserved across multiple species (Figure S1A), highlighting its potential functional importance. To directly assess the role of nuclear IDH3A in melanoma, we generated a construct in which the NLS was deleted (IDH3A‐dNLS), thereby disrupting its ability to translocate into the nucleus. Immunoblotting confirmed successful overexpression of IDH3A‐dNLS, and confocal immunofluorescence analysis showed that IDH3A‐dNLS was largely excluded from the nucleus (Figure S1B,D). Functional assays revealed that, in contrast to nuclear‐localized IDH3A constructs, IDH3A‐dNLS failed to promote melanoma cell proliferation, as demonstrated by colony formation and soft agar assays (Figure S1E,F), suggesting that the nuclear presence of IDH3A is critical for its pro‐tumorigenic function.

To directly assess the functional relevance of nuclear IDH3A, we engineered an IDH3A construct lacking its mitochondrial targeting sequence (IDH3A‐dMTS), thereby restricting its localization predominantly to the nucleus. Immunofluorescence analysis confirmed that this IDH3A‐dMTS localized mainly to the nucleus, in contrast to wild‐type protein, which resides in the mitochondria (Figure 3B,D).

In addition, we generated a catalytically impaired mutant (IDH3A‐mut), which harbors a point mutation that prevents its interaction with the IDH3β and IDH3γ subunits [17, 18], and thus cannot assemble into a functional IDH3 complex. Immunoblotting confirmed successful overexpression of both IDH3A‐dMTS and IDH3A‐ mut (Figure 3C). In addition, native PAGE analysis showed that the IDH3A‐mut exhibited a markedly reduced ability to assemble into the native IDH3 complex, suggesting impaired isocitrate dehydrogenase activity (Figure 3E).

Functional assays revealed that both IDH3A‐dMTS and IDH3A‐mut significantly enhanced cell proliferation, as evidenced by increased colony formation and anchorage‐independent growth in soft agar (Figure 3F,G). In vivo xenograft experiments further demonstrated that tumors derived from these modified cells exhibited accelerated growth compared to controls (Figure 3H). Immunohistochemical staining for Ki67 corroborated these findings, with higher proliferative indices observed in tumors overexpressing either IDH3A‐dMTS or IDH3A‐mut (Figure 3I).

Taken together, our results demonstrate that IDH3A can localize to the nucleus and promote melanoma progression through a mechanism independent of its traditional role as a subunit of isocitrate dehydrogenase.

### 3.4 IDH3A‐Associated Metabolic Remodeling is Dispensable for its Nuclear‐Driven Oncogenic Function

As a catalytic subunit of the isocitrate dehydrogenase 3 (IDH3) complex, IDH3A plays a pivotal role in the tricarboxylic acid (TCA) cycle, acting as one of its key rate‐limiting enzymes [19]. To further elucidate the impact of elevated IDH3A levels on melanoma metabolism, we conducted metabolomic profiling the MV3 melanoma cell line, focused on glucose metabolism and associated biosynthetic pathways.

**FIGURE 4:**
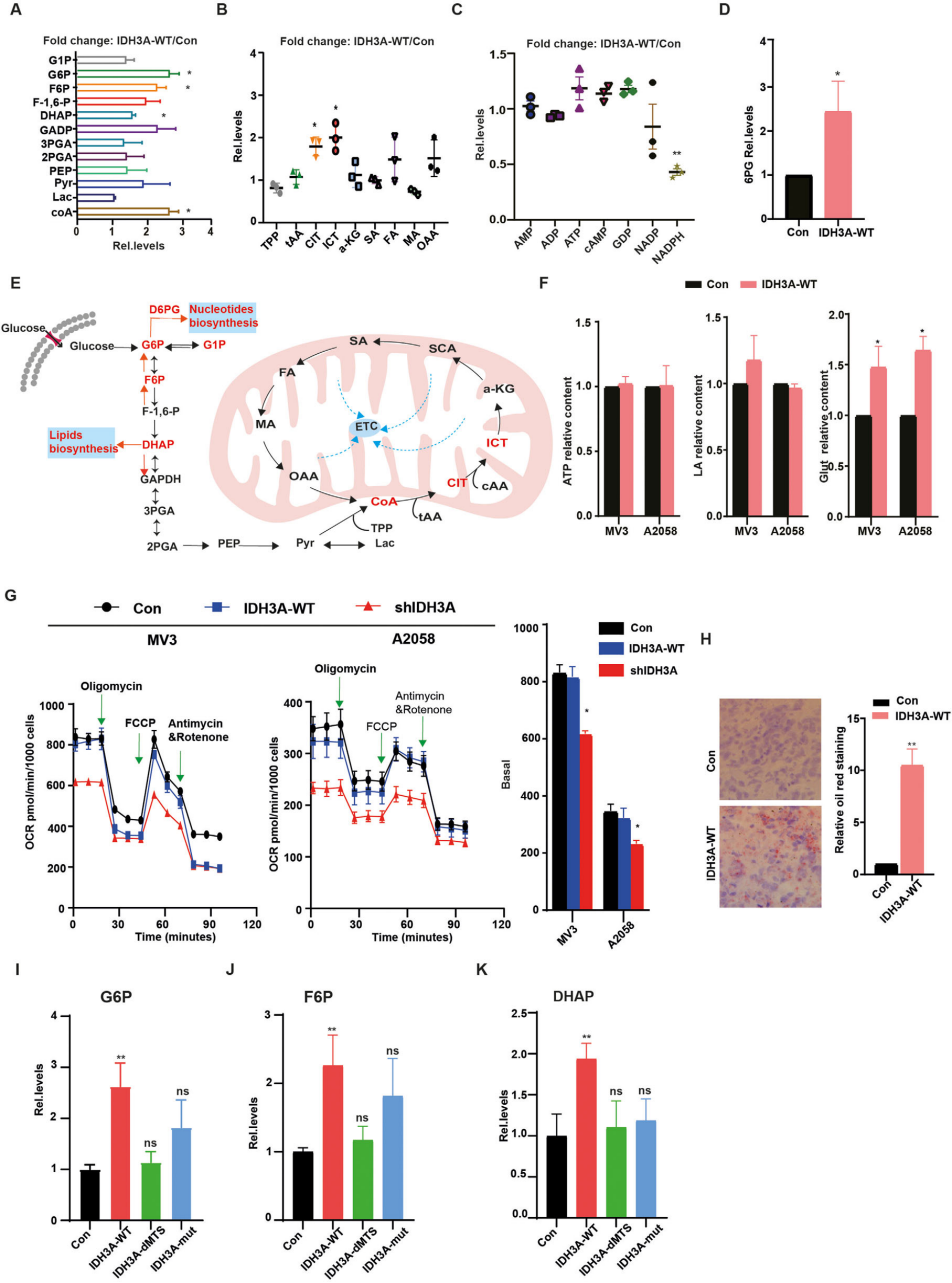
IDH3A‐associated metabolic remodeling is dispensable for its nuclear‐driven oncogenic function. (A–C) Untargeted metabolomic profiling showing glycolytic (A), TCA cycle (B), and nucleotide (C) intermediates in IDH3A‐overexpressing cells. (D) Targeted quantification of 6‐phosphogluconate (6PG). (E) Schematic overview of glycolysis and TCA pathway alterations upon IDH3A overexpression. (F) Intracellular ATP, lactate, and glucose levels upon IDH3A overexpression. (G) Oxygen consumption rate (OCR) in IDH3A‐overexpressing or knockdown cells. (H) Oil Red O staining of IDH3A‐overexpressing cells. (I) Intracellular G6P levels in cells expressing IDH3A, IDH3A‐mut, or IDH3A‐dMTS. (J) Intracellular F6P levels in cells expressing IDH3A, IDH3A‐mut, or IDH3A‐dMTS. (K) Intracellular DHAP levels in cells expressing IDH3A, IDH3A‐mut, or IDH3A‐dMTS.

Our data revealed that overexpression of IDH3A significantly increased intracellular concentrations of glucose‐6‐phosphate (G6P), fructose‐6‐phosphate (F6P), and dihydroxyacetone phosphate (DHAP)—intermediates in glycolysis that are essential precursors for nucleotide and lipid biosynthesis [20](Figure 4A). This suggests a shift toward anabolic metabolism to support rapid cell proliferation. Additionally, levels of NADPH, a crucial reductive cofactor for biosynthesis, were markedly reduced (Figure 4C). 6‐phosphogluconate (6PG), a product of the pentose phosphate pathway (PPP) and key substrate for nucleotide synthesis, was also increased in IDH3A‐overexpressing cells (Figure 4D).

Interestingly, within the TCA cycle, we observed elevated levels of Coenzyme A, citrate, and isocitrate, whereas the concentration of α‐ketoglutarate—the direct catalytic product of IDH3—remained unchanged (Figure 4B). This accumulation of upstream intermediates suggests a potential bottleneck or rerouting of carbon flux. Notably, isocitrate can serve as a substrate for transamination reactions, contributing to glutamate and aspartate synthesis, thereby facilitating amino acid and protein biosynthesis [21].

While the TCA cycle is traditionally associated with robust ATP generation [22], we did not observe a significant increase in intracellular ATP levels upon IDH3A overexpression (Figure 4F). Similarly, lactate production remained unchanged, suggesting that neither oxidative phosphorylation nor aerobic glycolysis (Warburg effect) was significantly enhanced (Figure 4F). However, glucose uptake was modestly increased in IDH3A‐overexpressing cells, indicating increased metabolic input despite unchanged ATP output.

To further examine mitochondrial respiratory function, we performed Seahorse extracellular flux analysis to assess oxygen consumption rates (OCR). Knockdown of IDH3A led to a notable reduction in basal OCR, confirming its role in maintaining mitochondrial metabolic activity (Figure 4G). In contrast, overexpression of IDH3A did not significantly enhance OCR (Figure 4G), possibly due to the complex regulation of the TCA cycle, which requires the concerted action of multiple rate‐limiting enzymes. These findings imply that IDH3A alone is not sufficient to drive full activation of mitochondrial oxidative metabolism.

Given the biosynthetic demands of rapidly proliferating cells, we next investigated lipid accumulation. Oil Red O staining revealed a marked increase in lipid content in MV3 IDH3A‐overexpressing cells (Figure 4H), suggesting enhanced fatty acid synthesis.

To determine whether these metabolic alterations were required for IDH3A‐mediated oncogenic activity, we compared the metabolic profiles of IDH3A‐WT with the IDH3A‐mut and IDH3A‐dMTS. Neither ATP production nor lactate secretion was significantly altered in cells expressing IDH3A‐mut or IDH3A‐dMTS, whereas both mutants exhibited increased glucose uptake compared with control cells (Figure S2A–C). Consistent with this observation, Oil Red O staining revealed enhanced lipid accumulation in both mutant‐expressing cells, suggesting that glucose utilization may be preferentially redirected toward anabolic biosynthetic pathways rather than canonical energy production (Figure S2D). We next performed targeted metabolite analysis of glycolytic intermediates. Interestingly, although IDH3A‐mut retained partial increases in glucose‐6‐phosphate (G6P) and fructose‐6‐phosphate (F6P) levels, it failed to reproduce the elevation of dihydroxyacetone phosphate (DHAP). Similarly, the dMTS mutant did not increase G6P, F6P, or DHAP levels despite maintaining tumor‐promoting activity (Figure 4I–K). These findings indicate that the oncogenic activity of nuclear IDH3A does not require full recapitulation of the metabolic remodeling induced by IDH3A‐WT and is largely independent of canonical IDH3 complex catalytic output.

Collectively, these results demonstrate that IDH3A overexpression induces an anabolic metabolic phenotype characterized by the accumulation of biosynthetic intermediates. However, the inability of IDH3A‐mut and IDH3A‐dMTS to fully recapitulate the metabolic alterations observed in IDH3A‐WT cells, despite retaining oncogenic activity, indicates that IDH3A‐mediated tumor promotion is primarily associated with its nuclear function rather than metabolic remodeling.

### 3.5 IDH3A Regulates c‐JUN and c‐FOS Expression Via Interaction with YBX1 in the Nucleus

**FIGURE 5:**
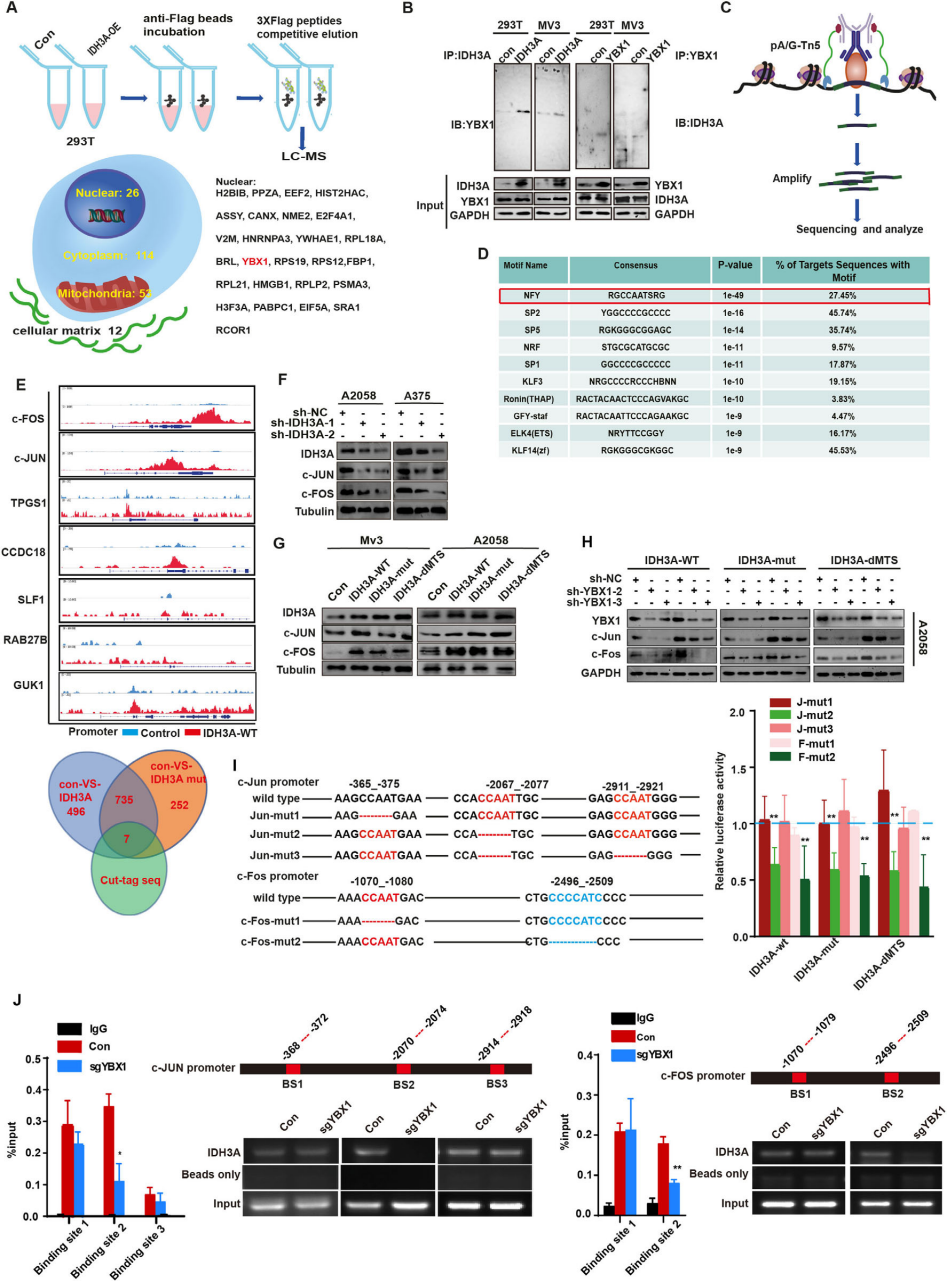
IDH3A interacts with YBX1 in the nucleus to regulate c‐JUN and c‐FOS expression. (A,B) Co‐immunoprecipitation (IP) and mass spectrometry identifying and validating the interaction between IDH3A and YBX1. (C,D) Schematic of the CUT&Tag workflow (C) and motif analysis of IDH3A‐binding sites (D). (E) Overlap between RNA‐seq differentially expressed genes (DEGs) and CUT&Tag targets in cells expressing IDH3A‐dMTS or IDH3A‐mut, together with CUT&Tag enrichment profiles of the seven overlapping genes. (F) Immunoblot analysis of c‐JUN and c‐FOS expression following IDH3A knockdown. (G) Immunoblot analysis of c‐JUN and c‐FOS expression in cells expressing IDH3A‐WT, IDH3A‐mut, or IDH3A‐dMTS. (H) Immunoblot analysis of YBX1, c‐JUN, and c‐FOS expression following YBX1 knockdown in A2058 cells expressing IDH3A‐WT, IDH3A‐mut, or IDH3A‐dMTS. (I) Schematic of wild‐type and mutant c‐JUN and c‐FOS promoter luciferase reporters and their relative luciferase activities in cells expressing IDH3A‐WT, IDH3A‐mut, or IDH3A‐dMTS. (J) ChIP‐qPCR and representative ChIP‐PCR analyses of IDH3A occupancy at the c‐JUN and c‐FOS promoters in control and YBX1‐knockdown cells.

To further elucidate the nuclear function of IDH3A, we overexpressed IDH3A in 293T cells and performed immunoprecipitation (IP) followed by mass spectrometry analysis. In addition to interacting with mitochondrial proteins, IDH3A was found to associate with cytoplasmic and nuclear proteins. Notably, among the nuclear interactors, we identified the transcription factor Y‐box binding protein 1 (YBX1) (Figure 5A). We validated this interaction by reciprocal co‐immunoprecipitation (co‐IP) assays in both 293T and MV3 melanoma cells further supported the interaction between IDH3A and YBX1 (Figure 5B). To further verify this interaction in situ, we performed proximity ligation assays (PLA), which revealed abundant PLA puncta in melanoma cells expressing endogenous IDH3A and YBX1, whereas minimal signals were detected in the negative control, further supporting their close intracellular association (Figure S3C). The IDH3A–YBX1 interaction remained detectable following Benzonase treatment, although the interaction signal was moderately reduced. These findings indicate that the association is not entirely dependent on nucleic acids, while nucleic acids may contribute to stabilization of the complex (Figure S3A).

To investigate whether IDH3A can associate with chromatin, we performed CUT&Tag sequencing in cells overexpressing IDH3A (Figure 5C). The results revealed a significant enrichment of IDH3A at genomic DNA regions, particularly within promoter regions (Figure S3B). Importantly, the most enriched motif identified from the CUT&Tag data was RGCCAATSRG, an extended Y‐box consensus sequence that represents a well‐established YBX1‐binding element commonly present in promoter regions [23] (Figure 5D). This finding suggests that nuclear IDH3A may cooperate with YBX1 to regulate gene transcription.

To identify downstream targets of nuclear IDH3A, we performed RNA‐sequencing on cells overexpressing either IDH3A‐WT or IDH3A‐mut. By intersecting the CUT&Tag‐enriched genes with the RNA‐seq–upregulated genes, we identified seven candidate targets: c‐FOS, c‐JUN, TPGS1, CCDC18, SLF1, RAB27B, and GUK1 (Figure 5E). Among these, c‐FOS and c‐ JUN showed the strongest CUT&Tag enrichment signals at their promoter regions (Figure 5E).

We next examined whether IDH3A regulates the expression of c‐FOS and c‐JUN. Western blot analysis demonstrated that both IDH3A‐WT, IDH3A‐dMTS and IDH3A‐mut significantly upregulated c‐FOS and c‐JUN expression, whereas IDH3A knockdown led to a notable decrease (Figure 5F,G). In contrast, overexpression of the nuclear localization‐deficient IDH3A‐dNLS mutant failed to alter c‐FOS or c‐JUN expression (Figure S1C).

To determine whether YBX1 mediates IDH3A‐induced c‐FOS and c‐JUN expression, we silenced YBX1 in IDH3A‐overexpressing cells. Western blot analysis showed that knockdown of YBX1 abolished the upregulation of c‐FOS and c‐JUN, indicating that the effect of IDH3A is YBX1‐dependent (Figure 5H).

To directly assess transcriptional regulation, we constructed luciferase reporter vectors containing the c‐FOS or c‐JUN promoter regions. Overexpression of IDH3A‐WT, IDH3A‐dMTS, or IDH3A‐mut significantly activated both promoters, confirming that IDH3A can function as a transcriptional coactivator independent of its dehydrogenase activity (Figure S3D). Knockout of YBX1 significantly reduced c‐JUN/c‐FOS promoter activity, whereas re‐expression of YBX1 restored reporter activity (Figure S3E). Consistently, immunohistochemistry (IHC) analysis of clinical melanoma specimens revealed a strong positive correlation between IDH3A and c‐FOS/JUN protein levels (Figure S3F).

Within the JUN promoter, we identified three putative YBX1‐binding motifs located at positions −365 to −375, −2067 to −2077, and −2911 to −2921. Site‐directed mutagenesis of these motifs revealed that mutation of the −2067 to −2077 site significantly impaired IDH3A‐mediated transcriptional activation, whereas mutations at the other two sites had no significant effect (Figure 5I). For the c‐FOS promoter, although a canonical YBX1 box was located at −1070 to −1079, its mutation did not affect IDH3A‐induced expression. Interestingly, we discovered a non‐canonical YBX1‐binding site (CCCCATC) at −2496 to −2509. Mutation of this site significantly reduced IDH3A‐induced c‐FOS promoter activity (Figure 5I), suggesting that this non‐classical element mediates IDH3A/YBX1 regulation of FOS transcription. ChIP–qPCR further confirmed the above results by demonstrating significant enrichment of IDH3A at the c‐JUN and c‐FOS promoters. This enrichment was markedly reduced following YBX1 depletion, indicating that YBX1 is required for efficient recruitment of IDH3A to these target loci (Figure 5J). To determine whether c‐JUN mediates the oncogenic effects of nuclear IDH3A, we depleted c‐JUN in IDH3A‐dMTS‐expressing melanoma cells. Knockdown of c‐JUN markedly attenuated the enhanced colony‐forming and anchorage‐independent growth induced by IDH3A‐dMTS in both A2058 and MV3 cells (Figure S3G,H), indicating that activation of c‐JUN is functionally required for the tumor‐promoting activity of nuclear IDH3A. Collectively, these findings demonstrate that nuclear IDH3A interacts with YBX1 to regulate transcription of c‐FOS and c‐JUN, two key transcription factors implicated in tumor proliferation and progression in melanoma.

### 3.6 NONO Regulates the Nuclear Localization and Oncogenic Function of IDH3A

To investigate the molecular mechanism underlying the nuclear localization of IDH3A, we performed immunoprecipitation (IP) followed by mass spectrometry (MS) using lysates from IDH3A‐WT, IDH3A‐dMTS, and control melanoma cells. Mass spectrometry analysis revealed a specific interaction between IDH3A and the RNA‐binding protein NONO in melanoma cells.

**FIGURE 6:**
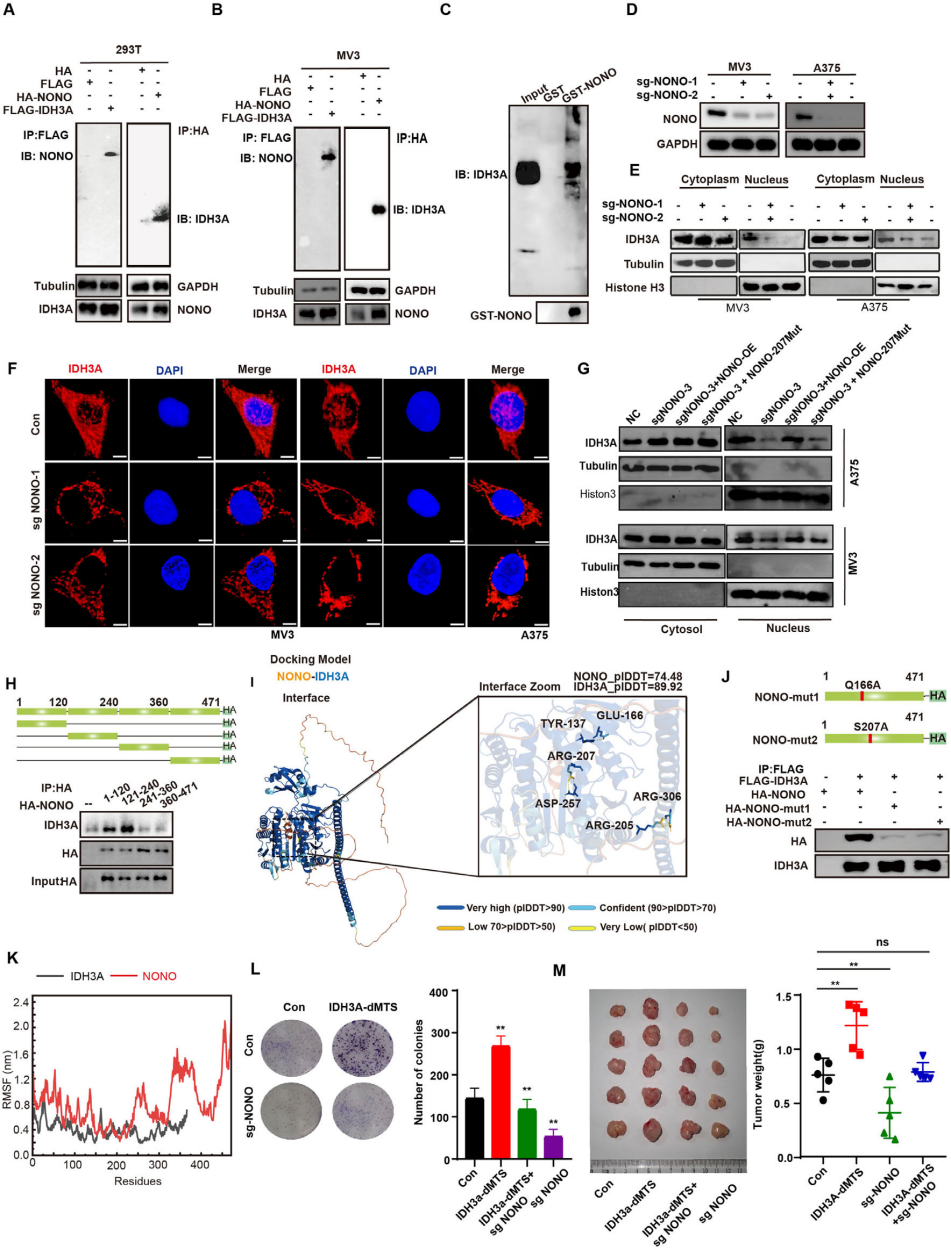
NONO regulates the nuclear localization and oncogenic function of IDH3A. (A) Reciprocal co‐immunoprecipitation of endogenous IDH3A and NONO in A375 cells. (B) Reciprocal co‐immunoprecipitation of endogenous IDH3A and NONO in MV3 cells. (C) GST pull‐down assay demonstrating the interaction between IDH3A and NONO. (D) Immunoblot analysis of NONO expression following NONO knockdown in MV3 and A375 cells. (E) Cytoplasmic and nuclear fractionation analysis of IDH3A following NONO knockdown in MV3 and A375 cells. (F) Representative immunofluorescence images showing the subcellular localization of IDH3A after NONO knockdown in MV3 and A375 cells. Scale bars: 10 µm. (G) Cytoplasmic and nuclear fractionation analysis of IDH3A following re‐expression of wild‐type NONO or NONO‐S207A in NONO‐deficient cells. (H) Mapping of the IDH3A‐binding region in NONO using HA‐tagged truncation mutants and co‐immunoprecipitation. (I) Structural model of the IDH3A–NONO interaction generated by molecular docking. (J) Co‐immunoprecipitation analysis of wild‐type and mutant NONO proteins with IDH3A. (K) RMSF profiles of IDH3A and NONO during molecular dynamics simulation of the IDH3A–NONO complex. (L) Representative colony formation assays and quantification following NONO knockdown in cells expressing IDH3A‐dMTS. (M) Representative xenograft tumors and quantification of tumor weight in mice implanted with cells expressing IDH3A‐dMTS with or without NONO knockdown.

To validate this interaction, we transiently co‐expressed IDH3A and NONO in both 293T and MV3 cells and performed IP assays. The results confirmed that IDH3A physically interacts with NONO (Figure 6A,B). To determine whether this interaction is direct, we conducted GST pull‐down assays, which confirmed direct binding between the two proteins (Figure 6C). Consistently, proximity ligation assays (PLA) revealed abundant punctate signals in melanoma cells, whereas minimal signals were observed in the negative control, further confirming the intracellular association between endogenous IDH3A and NONO (Figure S4F).

Next, to assess whether NONO regulates the nuclear localization of IDH3A, we generated NONO knockout MV3 and A375 cell lines using sgRNA‐mediated CRISPR/Cas9. Successful NONO depletion was confirmed by Western blot (Figure 6D). Nuclear‐cytoplasmic fractionation assays revealed a marked decrease in nuclear IDH3A upon NONO knockout (Figure 6E). Re‐expression of wild‐type NONO restored the nuclear localization of IDH3A in both A375 and MV3 cells (Figure 6G). Immunofluorescence using confocal microscopy further demonstrated reduced nuclear IDH3A signals in NONO‐deficient cells (Figure 6F).

To identify the region of NONO responsible for interacting with IDH3A, we generated a series of HA‐tagged NONO truncation mutants (HA‐NONO‐1–120, ‐121–240, ‐241–360, and ‐360–470) and performed co‐IP assays. The results showed that the 121–240 amino acid region of NONO exhibited the strongest binding to IDH3A, whereas the 1–120 region showed weak interaction, and the 241–470 regions did not interact at all (Figure 6H). Structure prediction by AlphaFold indicated a high pLDDT (pLDDT‐NONO = 74.48, pLDDT‐IDH3A = 89.92), pinpointing Gln166 (Q166) and Ser207 (S207) as key contact residues within the 121–240 region (Figure 6I). Molecular dynamics simulations further supported the stability of the predicted IDH3A–NONO complex. The RMSD profile reached equilibrium after approximately 40 ns, indicating that the complex adopted a stable conformation during the simulation (Figure S4B). RMSF analysis revealed limited fluctuations within the predicted binding interface, whereas the N‐ and C‐terminal regions of NONO displayed greater flexibility (Figure 6K). In addition, the complex maintained a stable number of hydrogen bonds throughout the simulation, averaging approximately 16 hydrogen bonds, consistent with persistent intermolecular interactions (Figure S4A). MM/PBSA analysis further demonstrated a favorable binding free energy (ΔG_total = −104.59 kcal/mol), indicating that formation of the IDH3A–NONO complex is thermodynamically favorable (Figure S4C). Site‐directed mutagenesis of NONO at these residues (NONO‐Q166A and NONO‐S207A) significantly reduced its binding affinity for IDH3A in co‐IP experiments, confirming their functional relevance in mediating the interaction (Figure 6J).

To further investigate the mechanism by which NONO regulates IDH3A nuclear localization, we generated an NLS‐deficient IDH3A mutant (dNLS). Unlike wild‐type IDH3A, the IDH3A‐dNLS failed to co‐immunoprecipitate NONO, indicating that the intrinsic NLS of IDH3A is required for its interaction with NONO (Figure S4D). Moreover, In contrast to wild‐type NONO, NONO‐S207A failed to restore nuclear accumulation of IDH3A in NONO‐deficient cells (Figure 6G). In contrast, CHX chase assays showed that NONO depletion did not significantly affect the stability of nuclear IDH3A (Figure S4E), suggesting that NONO facilitates the nuclear localization of IDH3A through direct interaction rather than by regulating its nuclear stability.

Functionally, NONO depletion significantly impaired the clonogenic capacity of melanoma cells and reversed the enhanced colony formation induced by IDH3A‐dMTS (Figure 6L). In vivo, NONO knockout abrogated the tumor‐promoting effect of IDH3A‐dMTS in subcutaneous xenograft models (Figure 6M). Taken together, these findings demonstrate that NONO is a critical regulator of IDH3A nuclear localization and its pro‐tumorigenic activity in melanoma.

### 3.7 Nuclear IDH3A Regulates Apoptosis in Melanoma Cells

**FIGURE 7:**
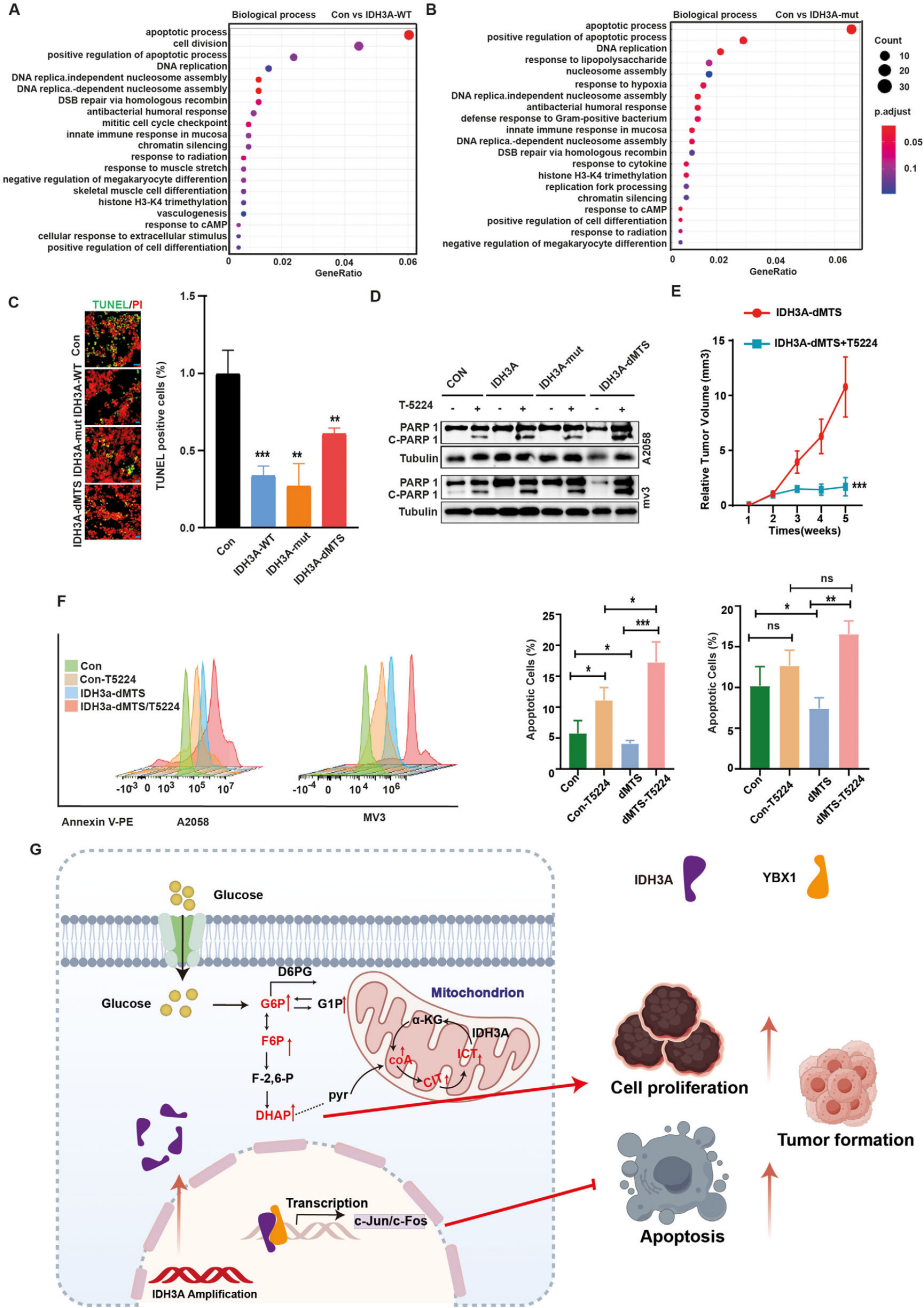
Nuclear IDH3A suppresses apoptosis in melanoma cells. (A) Gene Ontology (GO) biological process enrichment analysis of differentially expressed genes in cells expressing IDH3A‐WT. (B) Gene Ontology (GO) biological process enrichment analysis of differentially expressed genes in cells expressing IDH3A‐mut. (C) Representative TUNEL staining images and quantification of apoptotic cells in xenograft tumors. Scale bars: 10 µm (D) Immunoblot analysis of PARP cleavage in A2058 and MV3 cells following T‐5224 treatment. (E) Tumor growth curves of mice bearing IDH3A‐dMTS‐expressing tumors with or without T‐5224 treatment. (F) Flow cytometric analysis and quantification of Annexin V‐positive apoptotic cells following T‐5224 treatment in A2058 and MV3 cells. (G) Schematic model illustrating the oncogenic mechanism of nuclear IDH3A in melanoma.

To further elucidate the functional role of IDH3A in melanoma, we performed biological process enrichment analysis based on RNA‐seq data from cells overexpressing IDH3A‐WT and IDH3A‐mut. In addition to pathways associated with rapid cell proliferation, we observed significant alterations in apoptosis‐related signaling pathways in both groups (Figure 7A,B).

To validate these findings, we performed TUNEL staining on tumor sections derived from IDH3A‐WT, IDH3A‐mut, and nuclear‐localized IDH3A lacking the mitochondrial targeting sequence (IDH3A‐dMTS). Compared to the control group, all three experimental groups exhibited a marked reduction in apoptotic cells (Figure 7C), indicating that IDH3A expression suppresses apoptosis in melanoma.

To determine whether this anti‐apoptotic effect is mediated via the c‐FOS/JUN axis, we treated IDH3A‐WT, IDH3A‐mut, and IDH3A‐dMTS overexpressing cells with T‐5224, a pharmacological inhibitor of AP‐1 (c‐JUN/FOS) activity. Western blot analysis revealed that cleaved PARP, a hallmark of apoptosis, was significantly increased in both the IDH3A‐mut and IDH3A‐dMTS groups upon T‐5224 treatment, compared to untreated controls (Figure 7D).

In vivo, treatment with T‐5224 substantially reduced tumor growth in a subcutaneous xenograft model using IDH3A‐dMTS cells, further supporting the role of the IDH3A–c‐JUN/FOS axis in melanoma tumorigenesis (Figure 7E). Consistently, flow cytometry–based Annexin V/PI staining revealed a significant increase in apoptotic cells upon T‐5224 treatment in the IDH3A‐dMTS group in vitro (Figure 7F).

Collectively, these findings suggest that nuclear IDH3A promotes melanoma progression, at least in part, by suppressing apoptosis via the c‐JUN/FOS transcriptional program.

## 4 Discussion

In this study, we identify a previously unrecognized noncanonical role of IDH3A in promoting melanoma progression through nuclear translocation and transcriptional regulation. While IDH3A has been traditionally studied as a mitochondrial metabolic enzyme catalyzing oxidative decarboxylation in the TCA cycle [24], our data reveal that a fraction of IDH3A localizes to the nucleus in melanoma cells, where it acts independently of its enzymatic function to drive tumor growth. Mechanistically, nuclear IDH3A physically interacts with the transcription factor YBX1 and promotes transcription of c‐FOS and c‐JUN, two core components of the AP‐1 complex [25], thereby suppressing apoptosis and enhancing tumorigenicity (Figure 7G). We further demonstrate that the RNA‐binding protein NONO facilitates the nuclear import of IDH3A and is required for its oncogenic transcriptional function.

Our findings broaden the functional landscape of metabolic enzymes in cancer by uncovering a signaling role for IDH3A beyond mitochondrial metabolism. While nuclear localization of TCA‐cycle enzymes has been observed in embryogenesis and select cancer contexts [26], most notably fumarase and pyruvate dehydrogenase (PDH), our work adds IDH3A to the expanding list of “moonlighting” metabolic regulators that contribute to transcriptional control. Importantly, the tumor‐promoting function of nuclear IDH3A is independent of its canonical interaction with IDH3β/γ subunits and does not significantly alter cellular ATP or lactate levels, suggesting a complete decoupling from its metabolic enzymatic activity.

A particularly striking aspect of our study is the discovery that IDH3A enhances transcription of c‐FOS and c‐JUN by partnering with YBX1 on their promoter regions. AP‐1 is a well‐established oncogenic transcription factor complex in melanoma, regulating diverse processes including cell proliferation, survival, and metastasis [27]. By integrating CUT&Tag and RNA‐seq data, we show that nuclear IDH3A selectively co‐enriches with YBX1 on AP‐1 gene loci and functionally drives their expression. This cooperative regulation is further validated by promoter‐reporter assays and point mutations within YBX1‐binding sites, establishing a direct transcriptional role for IDH3A‐YBX1 axis. Interestingly, motif enrichment analysis also identified several additional transcription factor motifs, including NFY, SP‐family, KLF, NRF, and ETS family motifs. Although these motifs may indicate that the IDH3A–YBX1 complex participates in a broader transcriptional regulatory network, their functional relevance was not investigated in the present study and therefore warrants further investigation.

Our identification of NONO as a mediator of IDH3A nuclear localization adds another layer of regulation and provides a potential therapeutic vulnerability. NONO is known to function in RNA metabolism, transcriptional regulation, and nuclear organization, and has been implicated in multiple tumor types [28, 29]. Here, we show that NONO interacts directly with IDH3A via its 121–240 amino acid region, and mutations at residues Q166 and S207 diminish this interaction, reducing nuclear localization of IDH3A and attenuating its oncogenic function. The requirement of NONO for IDH3A‐driven tumor growth was further validated in vivo, highlighting a functional NONO‐IDH3A‐YBX1 axis that may be targetable in melanoma.

These findings challenge the classical view of IDH3A solely as a metabolic enzyme and instead position it as a nuclear co‐regulator of oncogenic transcription. Given the prevalence of IDH3A copy number amplification in melanoma and its correlation with poor prognosis, our study suggests that nuclear IDH3A may serve as both a prognostic biomarker and a potential therapeutic target. Moreover, the identification of YBX1 and NONO as functional partners offers new avenues for disrupting this transcriptional program in melanoma.

In summary, we uncover a nonmetabolic, nuclear function of IDH3A in melanoma and delineate a molecular mechanism by which it cooperates with YBX1 to promote oncogenic transcription and tumor progression. These findings provide a conceptual shift in how we understand metabolic enzymes in cancer biology and point to a multifaceted role of IDH3A that extends beyond energy metabolism into transcriptional regulation and tumor signaling.

## Author Contributions

**Juan Ran**: conceptualization, investigation, methodology, data curation, formal analysis, validation, visualization. **Yanchun Fang**: conceptualization, validation, methodology, data curation, resources. **Shengming Ruan**: conceptualization, methodology, data curation, formal analysis, validation. **Juling Wang**: validation, methodology, data curation. **Songbai Liu**: supervision, validation, resources. **Yuan Mao**: resources, data curation, methodology. **Dongsheng Hou**: validation, formal analysis, resources. **Yanping Wang**: validation, formal analysis, resources, writing – review and editing. **Ye Zi**: methodology, validation, investigation, writing – review and editing, formal analysis. **Qiong Li**: resources, validation, visualization, investigation, writing – review and editing. **Daoxiang Zhang**: conceptualization, methodology, data curation, supervision, resources, formal analysis, project administration, investigation, funding acquisition, writing – original draft, writing – review and editing, visualization.

## Funding

This study was supported by grants from the National Natural Science Foundation of China (82372682, 82073372).

## Conflicts of Interest

The authors declare no conflicts of interest.

## Supporting information




**Supporting File 1**: advs77762‐sup‐0001‐SuppMat.docx.

**Supporting File 2**: advs77762‐sup‐0002‐FigureS1‐S4.zip.

**Supporting File 3**: advs77762‐sup‐0003‐TableS1‐S2.zip.

## Data Availability

All material will be made available from the corresponding author upon reasonable request.
