# Supplementary material for: Nuclear IDH3A Drives Transcriptional Programs in Melanoma via the YBX1–JUN/FOS Axis
Source: Adv Sci (Weinh). 2026 Sep 11:e77762. Online ahead of print. doi: 10.1002/advs.77762 (PMC13564267; doi:10.1002/advs.77762)
Supplement: Supplementary file 1 [file ADVS-9999-e77762-s002.docx]

**Table S1.** **Primers used in this study.**

**Table S2.** **Key resources used in this study.**

**Supplementary Table 1**

**Figure S1. Nuclear localization of IDH3A is required for its tumor-promoting function.**
(A) Schematic representation of the predicted nuclear localization sequence (NLS) in IDH3α and its conservation across species.
(B) Immunoblot analysis showing the expression of NLS-deleted IDH3α in transfected cells.
(C) Immunoblot analysis of c-JUN and c-FOS levels upon overexpression of NLS-deleted IDH3α.
(D) Confocal immunofluorescence imaging showing subcellular localization of NLS-deleted IDH3A. Scale bars: 5 μm.
(E) Colony formation assay assessing the proliferative capacity of cells overexpressing NLS-deleted IDH3A.
(F) Soft agar assay evaluating anchorage-independent growth of cells expressing NLS-deleted IDH3A. Scale bars: 100 μm.

**Figure S2. Metabolic characterization of cells expressing IDH3A-dMTS or IDH3A-mut.**

(A) Intracellular ATP levels in cells expressing IDH3A-dMTS or IDH3A-mut.

(B) Intracellular lactate levels in cells expressing IDH3A-dMTS or IDH3A-mut.

(C) Intracellular glucose levels in cells expressing IDH3A-dMTS or IDH3A-mut.
(D) Oil Red O staining showing lipid accumulation in mutant-expressing cells.

**Figure S3. IDH3A–YBX1 interaction regulates c-JUN and c-FOS expression.**

(A) Reciprocal co-immunoprecipitation of IDH3A and YBX1 with or without Benzonase treatment.

(B) Genomic annotation of IDH3A CUT&Tag peaks.

(C) Representative PLA images showing the interaction between IDH3A and YBX1 in A375 and MV3 cells, with omission of the YBX1 antibody as a negative control. Scale bars: 5 μm.

(D) Relative luciferase activities of the c-JUN and c-FOS promoters in cells expressing IDH3A-WT, IDH3A-mut, or IDH3A-dMTS.

(E) Relative luciferase activities of the c-JUN and c-FOS promoters following YBX1 knockdown in cells expressing IDH3A-WT or IDH3A-dMTS.

(F) Correlation between nuclear IDH3A and c-FOS H-scores in melanoma tissues, with representative immunohistochemical staining of serial sections showing IDH3A, c-JUN, and c-FOS expression in tumors with low or high nuclear IDH3A levels.

(G) Representative colony formation assays and quantification following c-JUN knockdown in A2058 and MV3 cells expressing IDH3A-dMTS.

(H) Representative soft agar colony formation assays and quantification following c-JUN knockdown in A2058 and MV3 cells expressing IDH3A-dMTS. Scale bars: 100 μm.

**Figure S4. Supplementary analyses of the interaction between IDH3A and NONO.**

(A) Hydrogen bond analysis of the IDH3A–NONO complex during molecular dynamics simulations.

(B) Root mean square deviation (RMSD) analysis of the IDH3A–NONO complex during molecular dynamics simulations.

(C) MM/PBSA binding free energy decomposition of the IDH3A–NONO complex.

(D) Co-immunoprecipitation analysis of wild-type and NLS-deficient (dNLS) IDH3A with NONO.

(E) Cycloheximide (CHX) chase analysis of nuclear IDH3A following NONO knockdown.

(F) Representative proximity ligation assay (PLA) images showing the interaction between IDH3A and NONO in A375 and MV3 cells, with omission of the NONO antibody as a negative control. Scale bars:5 μm.
